# Ensemble detection of hand joint ankylosis and subluxation in radiographic images using deep neural networks

**DOI:** 10.1038/s41598-024-58242-0

**Published:** 2024-04-02

**Authors:** Keisuke Izumi, Kanata Suzuki, Masahiro Hashimoto, Masahiro Jinzaki, Shigeru Ko, Tsutomu Takeuchi, Yuko Kaneko

**Affiliations:** 1 Division of Rheumatology, Department of Internal Medicine, Keio University School of Medicine, Tokyo, Japan; 2 Medical AI Center, Keio University School of Medicine, Tokyo, Japan; 3 Division of Rheumatology, Department of Medicine, NHO Tokyo Medical Center, Tokyo, Japan; 4 AI Laboratories, Fujitsu Limited, Kanagawa, Japan; 5 Department of Radiology, Keio University School of Medicine, Tokyo, Japan; 6 Department of Systems Medicine, Keio University School of Medicine, Tokyo, Japan

**Keywords:** Computational biology and bioinformatics, Medical research, Rheumatology, Engineering

## Abstract

The modified total Sharp score (mTSS) is often used as an evaluation index for joint destruction caused by rheumatoid arthritis. In this study, special findings (ankylosis, subluxation, and dislocation) are detected to estimate the efficacy of mTSS by using deep neural networks (DNNs). The proposed method detects and classifies finger joint regions using an ensemble mechanism. This integrates multiple DNN detection models, specifically single shot multibox detectors, using different training data for each special finding. For the learning phase, we prepared a total of 260 hand X-ray images, in which proximal interphalangeal (PIP) and metacarpophalangeal (MP) joints were annotated with mTSS by skilled rheumatologists and radiologists. We evaluated our model using five-fold cross-validation. The proposed model produced a higher detection accuracy, recall, precision, specificity, F-value, and intersection over union than individual detection models for both ankylosis and subluxation detection, with a detection rate above 99.8% for the MP and PIP joint regions. Our future research will aim at the development of an automatic diagnosis system that uses the proposed mTSS model to estimate the erosion and joint space narrowing score.

## Introduction

The evaluation of joint destruction is essential in the clinical study of rheumatoid arthritis (RA) because joint destruction leads to difficulties in daily life activities. Particularly, the quantitative evaluation index for joint destruction based on the modified total Sharp score (mTSS^1^) is widely used in the RA clinical studies. The mTSS is always calculated by evaluating X-ray images of hands and feet at two different time points; the mTSS assessment of joint destruction is indicated by calculating a joint erosion score, which assesses bone damage, and a joint space narrowing (JSN) score, which assesses cartilage damage, in each of the joints that constitute the wrists, fingers and toes, and mTSS is expressed as the sum total of these joints. However, it requires the efforts of a skilled evaluator and therefore is seldomly used in daily clinical practice. In this study, we developed a method using deep neural network (DNN), which has shown remarkable performance in the medical field in recent years^2–4^, to detect the damaged joint regions required for automating mTSS evaluation.

To automate the estimation of mTSS, image processing approaches and machine learning approaches have been studied. In most commercial applications, despite their low versatility, image-processing approaches that recognise geometric features^5–7^ have been extensively utilised. If the application environment (e.g., the clinical site) changes, the image feature parameters must be redesigned accordingly. Conversely, in machine-learning approaches, particularly those using DNNs^8–11^, the image features required for recognition are automatically acquired during training, thus reducing the parameter design cost. The process of deriving the mTSS comprises the following steps.

- *Step 1* Detecting proximal inter phalangeal (PIP), inter phalangeal (IP), metacarpophalangeal (MP), and carpal joint regions.
- *Step 2* Classifying ankylosis, subluxation, and dislocation for each joint region.
- *Step 3* Classifying Sharp scores for each joint region.
- *Step 4* Calculating the mTSS from predicted each joint scores.

Although there are few previous studies on mTSS prediction using machine learning, there are some. Hirano et al.^8^ predicted the mTSS of PIP/IP and MP joint regions from an input image. However, the accuracy of the predicted score was not excessively high because of the peculiarities of the mTSS which diagnosis target is the finger deformation difference. Honda et al.^11^ reported that mTSS prediction accuracy can be improved by inputting two identical images with different augmentations into a CNN and introducing metric learning between the feature vectors. On the other hand, ankylosis, dislocation, and subluxation are classified as special findings, which score high according to the mTSS method (for wrists and fingers, Erosion 5 points and JSN 4 points for ankylosis, JSN 4 points for dislocation and JSN 3 points for subluxation). Murakami et al.^9^ constructed a model for classifying special findings using training images segmented by image processing. Special findings have a particular influential effect on the final mTSS because they are given a large score. Therefore, to improve the final mTSS accuracy, we believe that the prediction performance of Steps 1–2 is crucial.

In image recognition fields, detection of joint regions (Step 1), which is required for deriving the mTSS, is considered an object-detection task. Conventional studies outside the RA field have been conducted on DNN-based hand-detection methods. Bambach et al.^12^ proposed a dataset for hand-activity recognition and a detection framework using a DNN. Dadashzade et al.^13^ proposed a hand segmentation and gesture classification method using a convolutional neural network. This method has proven robust against lighting fluctuations and complex backgrounds without using depth information. Cai et al.^14^ demonstrated the generalisation of a trained DNN model to a new domain. Since the DNN-based method can automatically design image features, we believe that the above method for general hand images can also be applied to X-ray images in RA. In addition, recent detection models such as SAM segment images without labels^15,16^. After that, the mainstream approach is to use CLIP^17^ to measure the distance in the feature space between each detection area and the natural language expressing the detection target. Although these approaches have the potential to be applied to medical images^18^, detection of special finding labels contained in mTSS remains difficult.

This paper focuses on Steps 1–2, proposes a method for detecting PIP/IP and MP joint regions, and classifies special findings from X-ray input images. As the objective was to verify the basic model for mTSS estimation, we excluded the carpal joint owing to its difficult prediction. A previous study^10^ tested the accuracy of the classification of special findings in Step 2 within a limited prediction target. However, this method was developed to perform detection using an entire hand image, which is different from the actual mTSS calculation. Therefore, we extended the previous study^10^ by introducing an ensemble mechanism that includes multiple detectors, one for each special finding. In the mTSS prediction targeted in this paper, multiple labels of findings are attached to each joint. Each finding is diagnosed by independent criteria while measuring joint severity. In general object detection tasks, only one label is attached to the target object, so this setting is unique to mTSS prediction. The proposed method focuses on this, creates multiple detectors with different characteristics, and performs ensemble prediction, which is commonly used in machine learning, in hopes of improving overall performance. Furthermore, similar methods have not been proposed in the machine learning research in the rheumatoid joint field mentioned above^8,9,11^.

In addition, a DNN usually requires large quantities of annotated data. In related studies^13,14^, the experiments used a published dataset; however, such datasets are usually not available in the RA field. Therefore, the annotation tool developed in our previous study^10^ was extended by adding a function to annotate the joint regions. Because the annotation tool was specifically designed for electronic medical records, the training data were collected efficiently. The proposed model was experimentally verified using these collected data. Additionally, the quality of labels is high because multiple professional clinicians have annotated them. We believe the model examination using the original dataset and its results are highly reliable. The contributions of this study are summarized as follows:

- We developed and extended a dedicated RA annotation tool for data collection.
- We built and analyzed a unique dataset for mTSS prediction.
- We proposed a PIP/IP and MP joint detector using ensemble prediction.

## Methods

### Patients and X-ray images

The included patients with RA in this study met either the 1987 American College of Rheumatology (ACR) or 2010 European Alliance of Associations for Rheumatology/ACR classification criteria of RA. For our experiments, a dataset was prepared from the electronic medical record system of the Division of Rheumatology at Keio University Hospital.

We randomly extracted 260 X-ray images of 130 patients acquired in 2015–2016. Each patient’s record had a pair of X-ray images taken at two different time points that included both hands, producing 260 samples in total. We excluded patients with X-ray images of both hands at only one time point. The study protocol was approved by the Ethics Committee at Keio University (No. 20160316), and written informed consent was waived because of the retrospective design. The study was conducted in accordance with the Declaration of Helsinki.

### Definition of ankylosis, subluxation, and dislocation

- Ankylosis: Ankylosis is the abnormal stiffening and immobility of a joint due to fusion of the bones.
- Dislocation: Dislocation is the complete separation of the bones forming a joint.
- Subluxation: Subluxation is a partial dislocation of a joint in which the articular surfaces remain partially in contact.

### Data preparation

**Figure 1:**
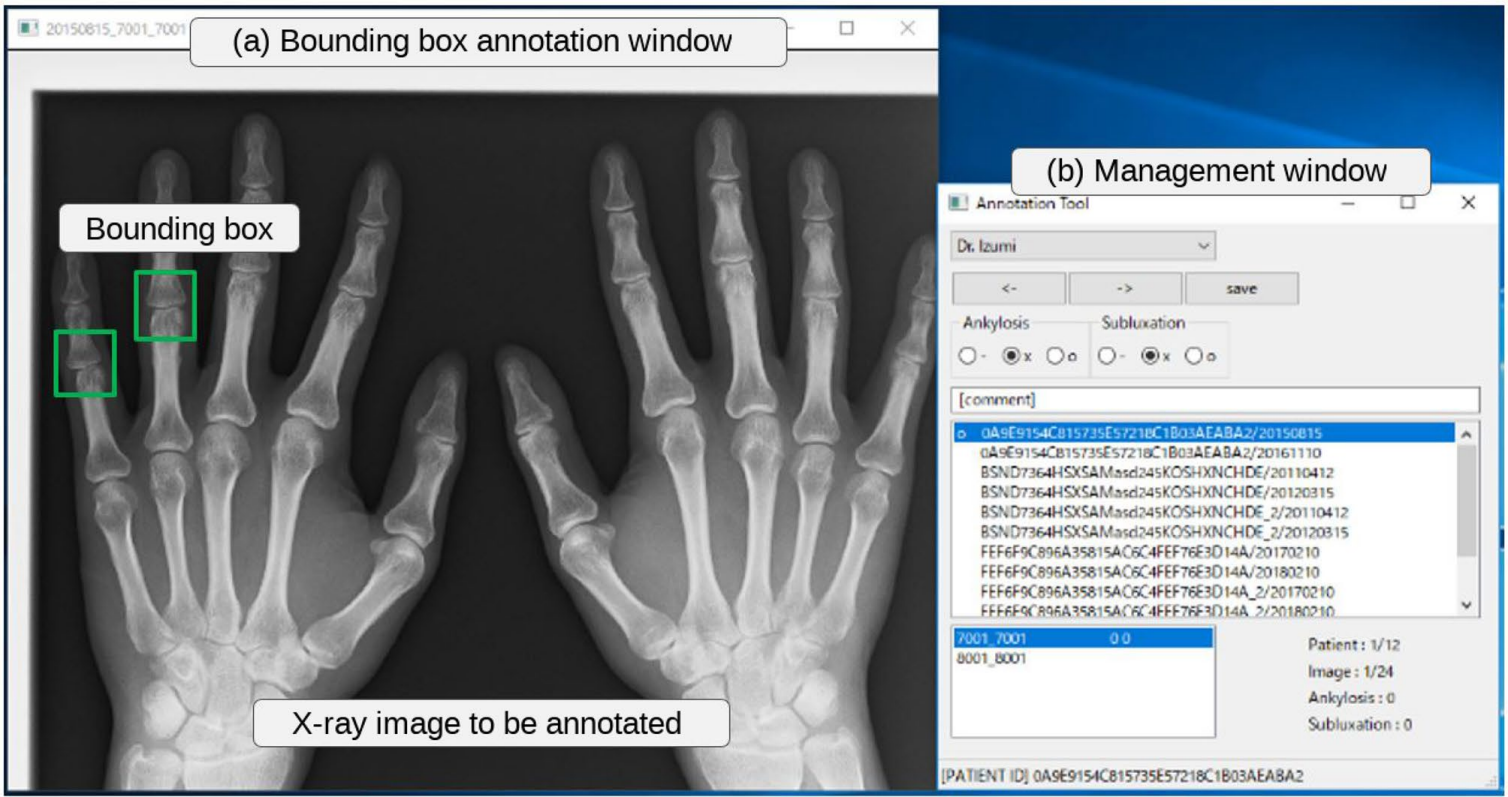
Developed annotation tool for the RA diagnostic system. The tool runs on an electronic medical record system in Keio Hospital, and it allows doctors to efficiently collect annotated data with bounding boxes and special findings in their spare time.

In DNNs, the quantity and quality of datasets are critical because they significantly affect the performance of the model. This subsection describes the construction of our dataset in the RA field. We annotated the PIP/IP and MP joints, excluding the carpal ones, in the extracted dataset using our developed annotation tool. Details on its graphical user interface are presented in Fig. 1. The user can select the image to be annotated from the dataset extracted from the electronic medical record system. The bounding box annotation window displays the X-ray image designated from the management window. The display screen can be enlarged, reduced, moved, and reset. The images were saved in the DICOM format using a $2010 \times 1670$ pixel resolution and 1024 gradations, which were specified as sufficient values for annotation by specialists. Annotation of the bounding boxes is performed using mouse drag-and-drop operations. The user annotates special findings on the displayed image by operating the management window. An annotation is recorded for each registered specialist (annotator) such that the user can suspend and resume the procedure at will. The tool was implemented on the electronic medical record system at Keio Hospital, enabling doctors to annotate efficiently.

The annotation position of the target joint is presented as a bounding box represented by the centre coordinates (*cx*, *cy*) and side lengths (*h*, *y*). Additionally, the target joints are annotated for the presence or absence of ankylosis, subluxation, and dislocation. Herein, subluxation and dislocation were assigned to the same label. As subluxation and dislocation have almost the same mTSS because it measures the difference between the before and after medication images. Predicting the exact differences separately has an insignificant effect on the final mTSS accuracy.

Annotation was conducted in conjunction by three well-trained rheumatologists and radiologists. Twenty PIP/IP and MP joints were annotated for each image. Labels for ankylosis and subluxation/dislocation were annotated on 20 and 18 bounding boxes, respectively, excluding the IP joint of the thumb. The annotator was not informed of the patients’ general and clinical information and diagnosis results to ensure that the results were based solely on imaging analysis.

### Development of the joint region detector using a DNN

**Figure 2:**
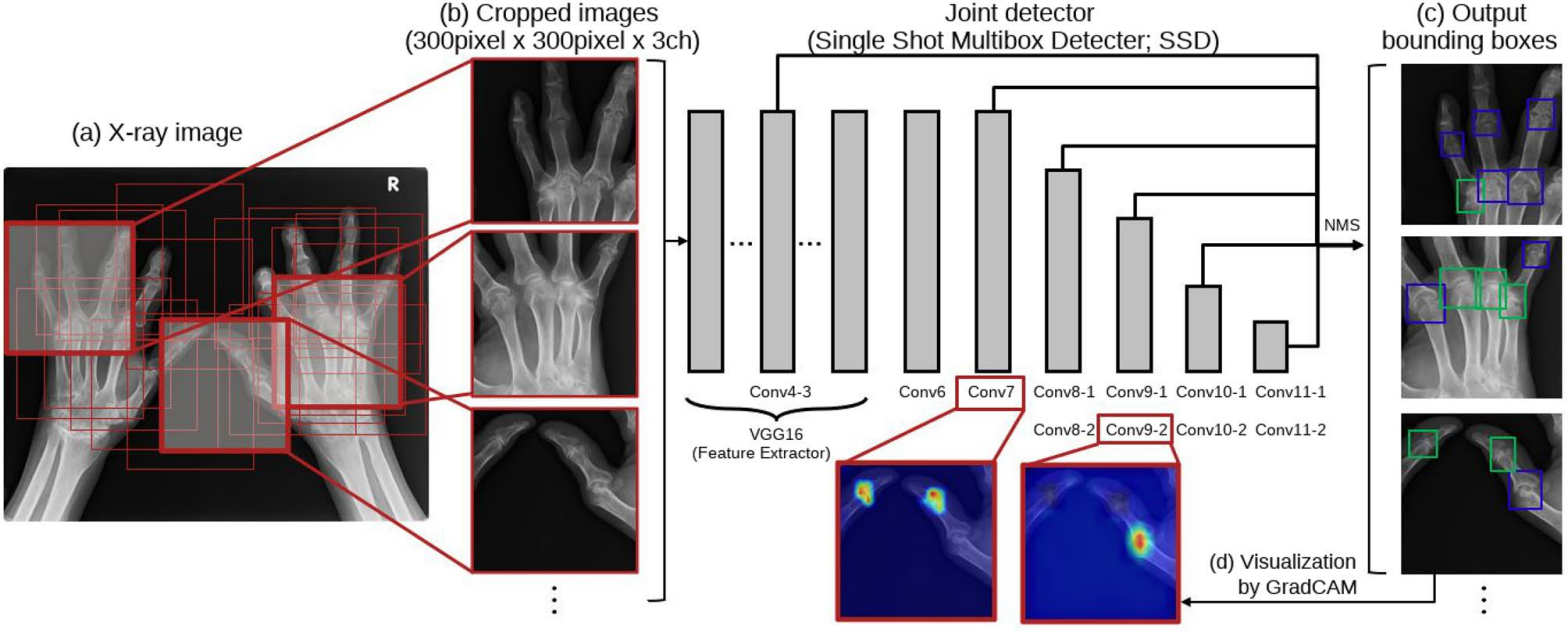
Overview of the learning process of the joint region detector (SSD^19^). Cropped X-ray images were used as inputs for SSD training to predict the position and size of multiple bounding boxes and associated class labels.

We developed a detector for the PIP/IP and MP joints to classify their special findings. We used a single shot multibox detector (SSD^19^), namely, SSD 300 with an input size of $300 \times 300 \times 3$ pixel, as the detection model. Figure 2 presents an overview of the SSD training process. An SSD is an object-detection DNN model comprising multiple convolution layers that predicts the position, size, and associated class labels of multiple bounding boxes in an end-to-end manner from the input X-ray image. The SSD incorporates VGG16^20^ as a feature extractor. Several convolutional layers (Conv 4, 7, 8, 9, 10, and 11) in the subsequent stage are connected to the output layer. The SSD detects objects of various sizes by preparing appropriately sized default bounding boxes in advance.

We trained the SSD for each ankylosis and subluxation/dislocation detection task. The total loss *L* for each SSD is calculated by combining the localisation loss $L_{loc}$ for the position of the bounding box and confidence loss $L_{conf}$ for the spcial finding class. The total loss *L* is defined as follows:

1 $$\begin{aligned} L(x,c,l,g)= & {} - \frac{1}{N} \left( L_{conf}(x,c) \right) + L_{loc}(x,l,g), \end{aligned}$$

2 $$\begin{aligned} L_{loc}(x,l,g)= & {} \sum _{i\in Pos}^N \sum _{m\in \{cx,cy,w,h\}} x_{ij}^k smooth_{L1} \left( l_i^m - {\hat{g}}_j^m \right) , \end{aligned}$$

3 $$\begin{aligned} L_{conf}(x,c)= & {} -\sum _{i\in Pos}^N x_{ij}^plog \left( {\hat{c}}_i^p \right) - \sum _{i\in Neg} log \left( {\hat{c}}_i^0 \right) \hspace{2mm} where \hspace{2mm} {\hat{c}}_i^p=\frac{\exp \left( c_i^p \right) }{\sum _p \exp \left( c_i^p \right) }, \end{aligned}$$

here $smooth_{L1}$^21^ and softmax loss are adopted to represent $L_{loc}$ and $L_{conf}$, respectively. *N* denotes the number of matched default boxes; $x_{ij}^p$ is an indicator of the match between the *i*-th default bounding and *j*-th ground-truth boxes of the *p* category; $c: (c_1,c_2,\dots c_p)$ denote the predicted class confidences; *l* and *g* denote bounding box predicted dimensions and ground-truth values that consist of (*cx*, *cy*, *w*, *h*). *Pos* and *Neg* denote the set of classes of the special findings and background, respectively. As in a previous study^22^, the model regressed the offsets of the center position (*cx*, *cy*), width *w*, and height *h* of the default bounding box *d* using the following conversion formula:

4 $$\begin{aligned} {\hat{g}}_j^{cx}= \left( g_j^{cx}-d_i^{cx} \right) /d_i^w, \hspace{5mm} {\hat{g}}_j^{cy}= \left( g_j^{cy}-d_i^{cy} \right) /d_i^h, \end{aligned}$$

5 $$\begin{aligned} {\hat{g}}_j^w=\log \left( g_j^w/d_i^w \right) , \quad {\hat{g}}_j^h=\log \left( g_j^h/d_i^h \right) . \end{aligned}$$

By minimising the aforementioned loss function, the model optimises the parameters to predict the bounding box position in the input image and class probabilities of the special findings for each box. Each SSD classifies the special findings using three classes indicating presence, absence, and the background.

We resized the images in the dataset to 50% of their original sizes (Fig. 2a) to ensure that the annotated bounding box was large enough to be predicted by SSD 300. The input image was prepared by cropping a $300 \times 300$ pixel window from the image at a position including at least one annotated bounding box (Fig. 2b). The entire hand image was not used as input in our study because RA patients often have severely deformed hands, which adversely affects learning. At every epoch, each joint in the image was randomly cropped. Therefore, 18 and 20 cropped images were created for each subluxation/dislocation and ankylosis joint detector image, respectively, during training. More detailed training parameters are described in the experimental setup chapter.

### Ensemble mechanism

**Figure 3:**
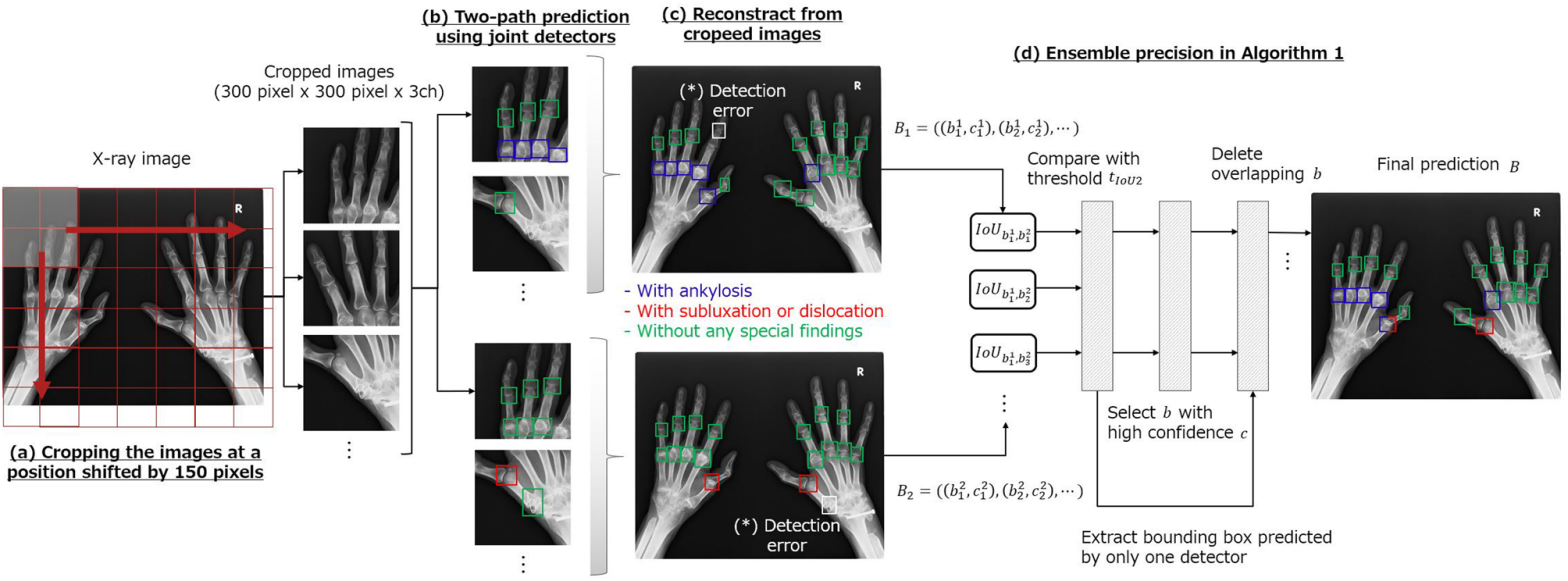
Overview of the proposed prediction process. By integrating the outputs of the ankylosis and subluxation/dislocation detectors through ensemble processing, the overall prediction performance was improved.

Next, we describe the proposed prediction process presented in Fig. 3. We used ensemble prediction to improve the overall detection performance of the proposed model. The general ensemble method trains multiple weak predictors and determines the final prediction by a majority vote^23^, complementing the performance of each model and enabling the bias and variance of the model to be reduced and adjusted evenly. The combination of predictors with different characteristics is particularly effective. In the proposed model for mTSS estimation, ensemble prediction was applied by constructing multiple predictors for the subluxation/dislocation or ankylosis classification tasks.

#### Individual SSD predictions

Before explaining ensemble prediction process, we will first explain the prediction process of individual SSDs. We constructed SSDs for each ankylosis and subluxation/dislocation detection task, and each model predicts the bounding box position in the input image and class probabilities of the special findings for each box. During evaluation, each detection model scans the entire X-ray image while shifting the input image by 150 pixels (Fig. 3a). The areas exceeding the X-ray image during shifting and cropping are filled with zero padding.

After scanning the entire image, all outputs are combined (Fig. 3c). Each output bounding box has a predicted class probability *c*, confidence score ${\hat{c}}=max(c)$, and predicted position and shape in the image *b* : (*cx*, *cy*, *w*, *h*), where *b* is restored from the predicted offset and default bounding box size (Eqs. 4–5). To remove duplicate bounding boxes, we used non-maximum suppression (NMS^24^). First, we extract only the default bounding boxes with confidence scores above the threshold $t_{conf}$. Subsequently, we calculate the intersection over union (IoU) between the predicted bounding boxes. If IoU is equal to or greater than the threshold $t_{IoU1}$, the bounding box with a low confidence score is removed. We set $t_{conf}=0.9$ and $t_{IoU1}=0.15$. Typically, the hand X-ray image used for mTSS estimation shows that the joint region’s bounding boxes minimally overlap. Therefore, in the proposed method, the threshold $t_{IoU1}$ is set to a strict value. *M* denotes the number of target joints contained in the image. The top *M* detection candidates with high confidence scores are selected from the remaining bounding boxes and used as the output of individual SSDs. We set $M_1=20$ and $M_2=18$ for the ankylosis and subluxation/dislocation detectors, respectively, to predict the target joints excluding the carpal ones.

#### Ensemble prediction

Finally, we show ensemble prediction process in Algorithm 1. In the proposed method, the aforementioned prediction process was applied to the individual SSDs of the special findings. The bounding boxes $B^1$ and $B^2$ are the outputs of each SSD using two-path prediction (Fig. 3b and lines 1–2 in Algorithm 1). When the two detectors are combined, ($M_1+M_2$) bounding boxes are predicted for each image.

**Algorithm 1:**
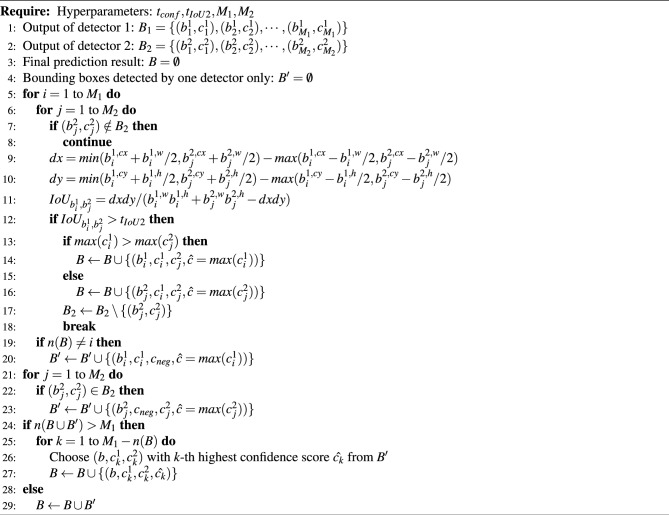
Proposed ensemble prediction procedure

First, we compare the prediction results of each detector, and the bounding boxes with IoUs above the threshold $t_{IoU2}$ are removed using NMS, assuming that they indicate the same finger joint region (lines 5–18 in Algorithm 1). Herein, we set $t_{IoU2}=0.45$. In terms of processing, we group the bounding boxes that satisfy the conditions are grouped into a set *B* that summarizes the prediction results. And, the detected bounding boxes are then given two special labels, $c^1$ and $c^2$ (lines 14 and 16 in Algorithm 1). The ones that IoU scores of less than $t_{IoU2}$ are assigned to a negative class probability $c_{neg}$ about the special finding and stocked in a temporary set $B^{\prime}$ (lines 20 and 23 in Algorithm 1). The above process extracts duplicate bounding boxes to *B* and unique bounding boxes to $B^{\prime}$.

At last, if there are ($M_1+1$) or more remaining candidates in $B\cup B^{\prime}$, selections are made in descending order of the confidence score until the number of bounding boxes reaches $M_1$. This is because at least one SSD output joint other than the PIP/IP and MP joints is included in $B\cup B^{\prime}$ (lines 24–29 in Algorithm 1). Through this process, the final prediction result *B* is obtained by complementing the two detector predictions.

## Experiments

### Training setup

This section describes the details of the experimental settings used in this study to test and evaluate the trained DNN model. We built the DNN models using Pytorch which is a machine learning framework. And, all other programs, including the annotation tools, are created using Python. The two ankylosis and subluxation/dislocation detection models were trained using datasets comprising 260 X-ray images extracted from electronic medical records. The images collected for ankylosis classification include 5200 PIP/IP and MP joints, of which 157 present ankylosis findings, while those collected for subluxation/dislocation classification include 4680 PIP and MP joints, of which 60 joints present subluxation/dislocation findings.

**Figure 4:**
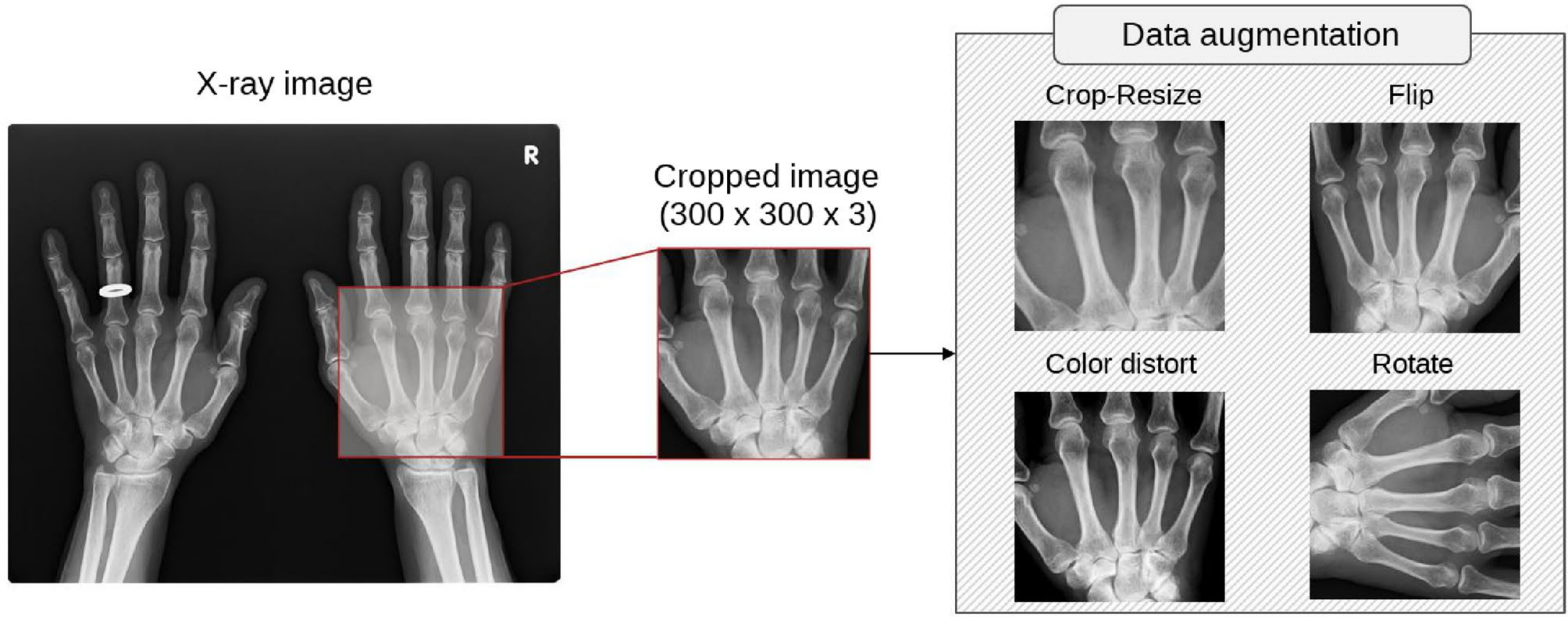
Input image pre-processing during the training phase. The X-ray image is randomly cropped as an input image for the SSD and is augmented by coloring, crop-resizing, rotating, and flipping.

Figure 4 shows the procedure for input image pre-processing during the training phase. X-ray images were converted to color images and used as input for the model. The structure of the SSD is optimized for RGB general images, and, in our preliminary experiments, we confirmed that learning progresses more smoothly when using color images. The input images were also augmented by colouring, crop-resizing, rotating, and flipping to increase model robustness. Augmentation was performed with a 50% probability, and the Adam optimiser^25^ was used for training, with parameters $\alpha =0.001$, $\beta _1=0.9$, $\beta _2=0.999$, and weight decay $=0.0005$. We did not use published pre-trained SSD weights owing to the domain differences between medical and other images. We trained the model for 200 epochs with a batch size of 16.

Five-fold cross-validation was conducted in the training phase. The master dataset was divided into five numbered fold sets, each with 210 training and 50 test images. The images with ankylosis or subluxation/dislocation findings were equally divided in each fold dataset. However, in few fold sets, the total number of special findings was not evenly represented owing to the presence of multiple findings in individual images.

### Evaluation

We evaluated the performance of the proposed model based on the accuracy, recall, precision, specificity, F-value, and IoU between the predicted bounding box and ground truth using the average results of the fold sets. We investigated the detection rate of the PIP/MP joint regions for each model and classification rate of the special findings in each region in the test dataset. During evaluation, detection was considered successful when the IoU between the bounding box and ground truth was above 0.45. The threshold value of IoU used in this study was set based on existing studies on general image detection tasks^19^. In the later process assumed in this study (diagnosis of findings by comparing two cropped images), the detection target can be included by cropping the image to a size larger than the predicted detection box. We believe that this will produce an output of sufficient quality for mTTS calculations as long as the above threshold of IoU is met.

Additionally, we visualised the regions contributing to the output using gradient-weighted class activation mapping (GradCAM^26^). The SSD layers visualised were Conv 7 and 9-2 (Fig. 2d). GradCAM computes the contributing regions of the input image based on gradient information from each of the DNN layers, as follows:

6 $$\begin{aligned} \alpha ^k_c= & {} \frac{1}{Z}\sum _i \sum _j \frac{\partial y_c}{\partial A^k_{i,j}}, \end{aligned}$$

7 $$\begin{aligned} G_c= & {} ReLU \left( \sum _k \alpha ^k_c A^k \right) , \end{aligned}$$

where *Z* denotes the number of pixels in the feature map *A*, *i* and *j* are pixel positions, *k* and *c* denote the channels and class index, respectively. The weight $\alpha$ of the feature map *A* is calculated from the gradient information obtained by backpropagation (Eq. 6). Any gradient other than the output target class was set to 0. The contributing regions $G_c$ of class *c* were determined by calculating the weighted sum of $\alpha$ and *A* (Eq. 7). The GradCAM calculation ignores the negative values; thus, only positive gradients affect the class. Because a quantitative evaluation of the basis for the output of the DNN model is difficult, we investigated whether the contributing regions are consistent with the corresponding medical diagnosis.

## Results and discussion

### Detection performance

Table 1 lists the detection accuracy results for the PIP/IP and MP joint regions of the test dataset for each fold set, with each column presenting the results of the model using ankylosis or subluxation/dislocation training and the proposed ensemble prediction. In a cascaded mTSS prediction system, missed joint region detections adversely affect the final score because subsequent processing cannot provide compensation. The ensemble prediction showed improved accuracy for all fold sets compared to those obtained using individual detection models, indicating that if one SSD failed during detection, the other could compensate for it. Therefore, it is expected to play a crucial role in improving the performance of the entire system.

**Table 1:** Detection results of single detector and the proposed method. Each column presents the detection rates [%] of the model using ankylosis or subluxation/dislocation training and the proposed ensemble prediction.

	Fold 1 (%)	Fold 2 (%)	Fold 3 (%)	Fold 4 (%)	Fold 5 (%)	Average (%)
Ankylosis	99.7	99.8	99.8	99.9	100.0	99.88
Subluxation	99.89	99.89	100.0	99.89	100.0	99.93
Ensemble	100.0	99.90	100.0	100.0	100.0	99.98

**Figure 5:**
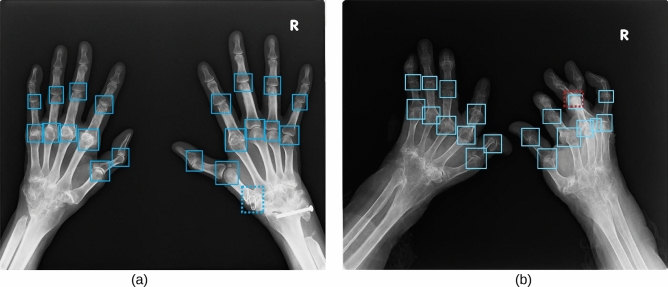
Examples of detected PIP/IP and MP joint regions using the proposed method. In (**a**), metal fittings embedded in the hand cause false detection when using the single detection model; the detection performed by the proposed ensemble method was successful. In (**b**), patient’s hand deformation and joint overlap impaired posterior joint detection. (**a**) Success case (**b**) Failed case.

The blue boxes in Fig. 5 show examples of the PIP/IP and MP joint regions detected using the proposed method. The blue dashed box indicates the joint region erroneously detected by individual SSDs, while the red one indicates those not detected using our method. Figure 5a shows an example of joint regions that are difficult to detect using image-processing methods that capture geometrical features or using a single detection model. Although metal fittings embedded in the hand caused false detections with other models, the proposed ensemble method accurately detected joint regions. Figure 5b shows the only case where detection using the proposed method was unsuccessful; specifically, the extremely deformed patient’s hand with overlapping joints prevented detected of the posterior one. In future, this aspect could be addressed by removing the overlap of the bounding boxes using a hand model prepared in advance.

### Classification performance

#### Subluxation and dislocation

Table 2 shows the classification results for subluxation and dislocation using the proposed model. Each classification result adopts the bounding box label predicted by the individual SSDs (see “Method” section). The average value of all fold-set results was 0.99 for accuracy, 0.81 for precision, 0.78 for recall, 0.99 for specificity, and 0.78 for the F-value. The average IoU between the detected bounding box and the ground truth was 0.902. Considering that images with subluxation or dislocation accounted for approximately 1–2% of the total training dataset, we can state that the classification accuracy was high. This suggests that the shape of the dislocated hand may be easily distinguished from the normal condition because of the significant change in the overall shape and finger postures. Our model accuracy could still be improved, and more data will be collected for this purpose in future research.

**Table 2:** Classification results of the proposed method for subluxation/dislocation. Each column presents the classification results [Num] of the model using subluxation/dislocation training.

	Fold 1	Fold 2	Fold 3	Fold 4	Fold 5
True positive	6	9	17	8	8
False positive	2	2	3	5	0
True negative	887	889	880	882	889
False negative	5	1	0	3	3

TP, FP, TN, and FN mean True Positive, False Positive, True Negative, and False Negative, respectively.

#### Ankylosis

Table 3 summarises the ankylosis classification results obtained using the proposed method. The average values of all fold-set results were 0.99, 0.98, 0.81, 0.99, and 0.88 for accuracy, precision, recall, specificity, and the F-value, respectively. The average IoU between the detected bounding box and ground truth was 0.907. These results showed higher classification accuracy than the subluxation and dislocation classification results, because the training dataset of the ankylosis detection model included more images with positive labels of special findings. This difference in the models can effectively improve each other results in our ensemble method.

**Table 3:** Classification results of the proposed method for ankylosis.

	Fold 1	Fold 2	Fold 3	Fold 4	Fold 5
True Positive	24	24	28	27	24
False Positive	1	0	1	1	0
True Negative	965	967	969	969	969
False Negative	8	8	3	4	7

Each column presents the classification results [Num] of the model using ankylosis training. TP, FP, TN, and FN mean True Positive, False Positive, True Negative, and False Negative, respectively.

### Visualisation

**Figure 6:**
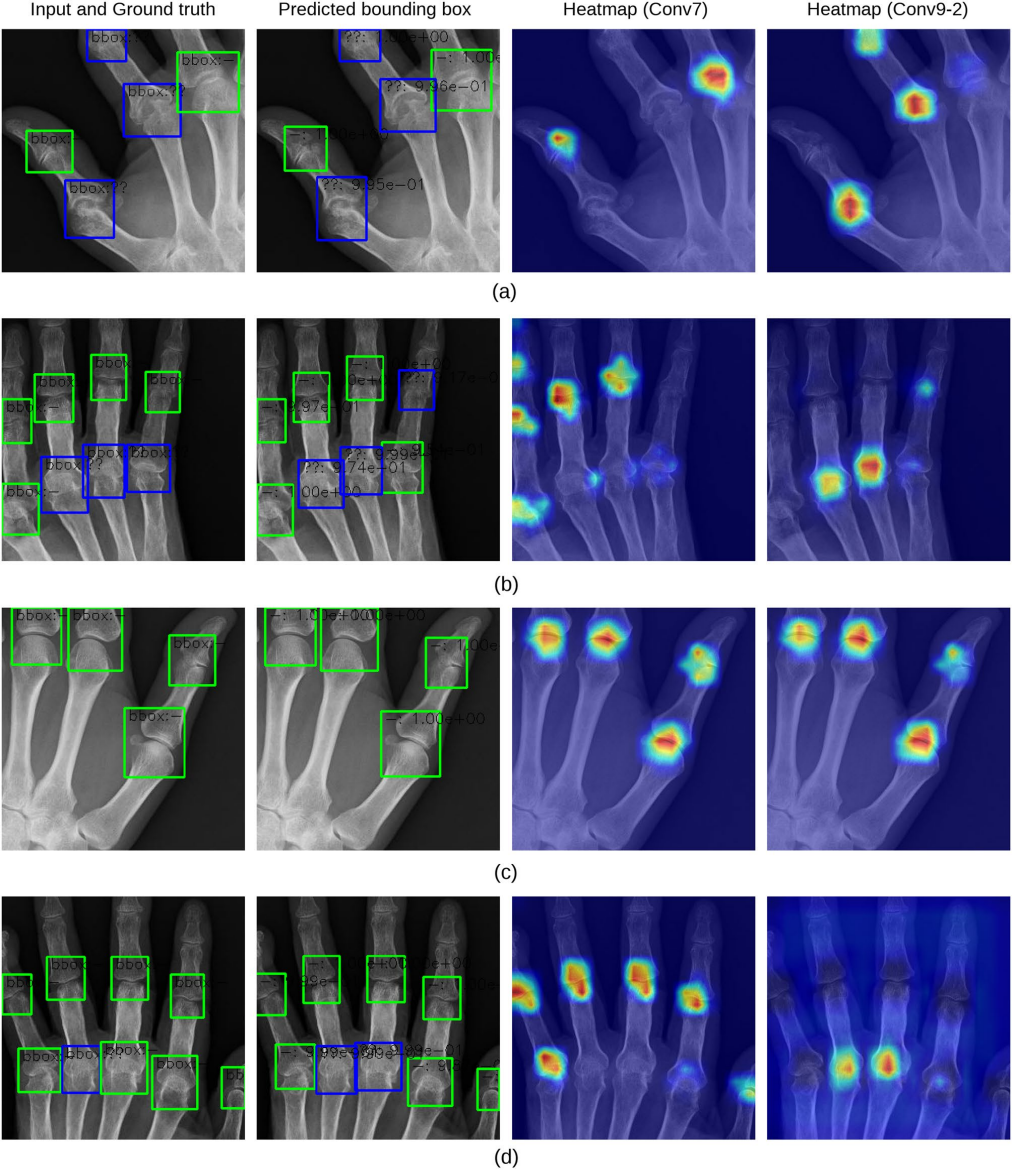
Prediction results of subluxation/dislocation in PIP/IP and MP joints and GradCAM visualization examples. The SSD often reacted to cover the entire joint regions. (**a**) True positive (**b**) False positive (**c**) True negative (**d**) False negative.

**Figure 7:**
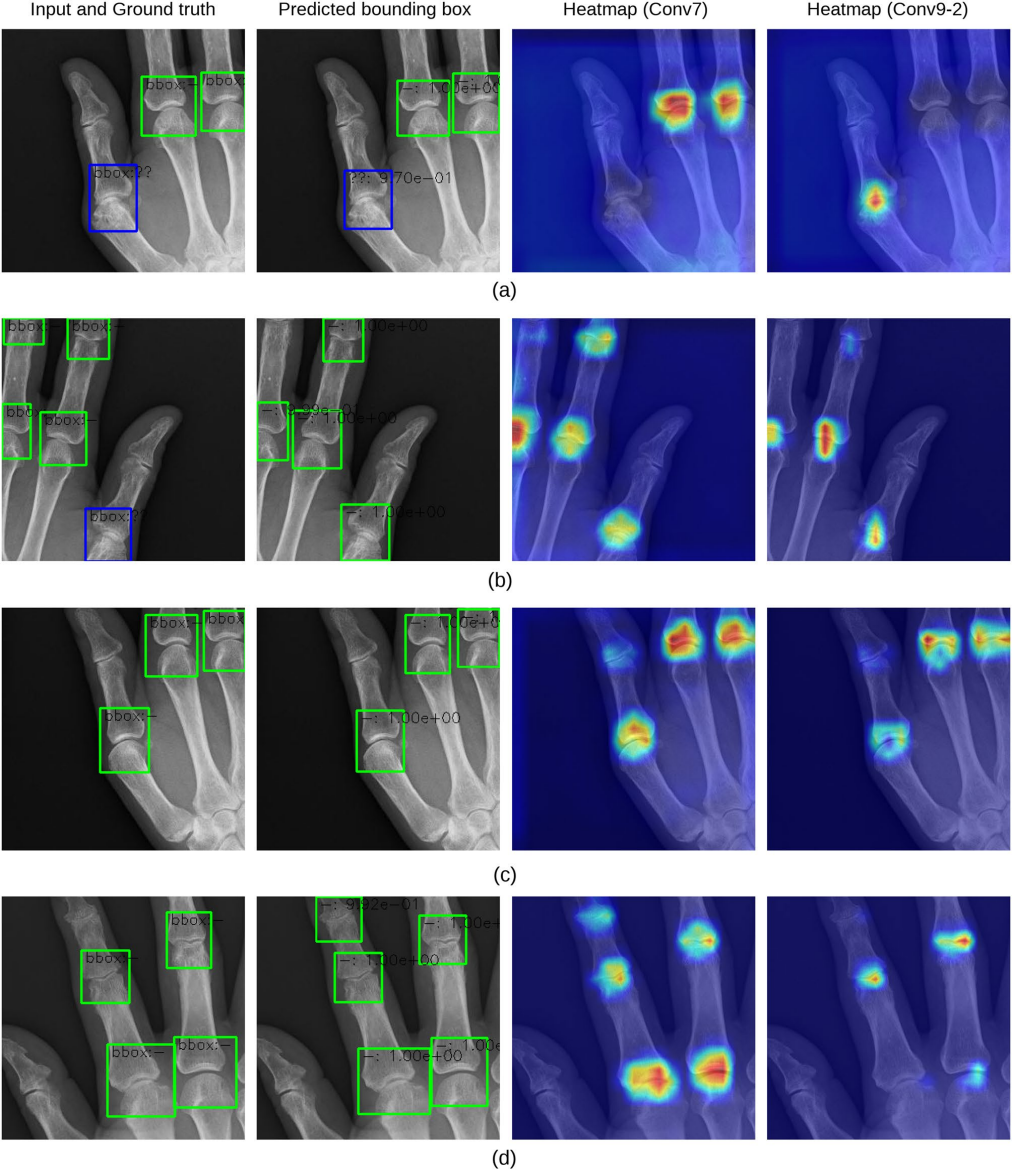
Prediction results of ankylosis in PIP and MP joints and GradCAM visualization examples. The SSD reacted strongly around the base of the joint. (**a**) True positive (**b**) False positive (**c**) True negative (**d**) False negative.

Herein, we present few examples of detected bounding boxes and heat maps for the visualisation of SSD contribution regions. The image pairs in Figs. 6 and 7 indicate typical examples of true positives, false positives, true negatives, and false negatives in the ankylosis or subluxation/dislocation classification tasks. The left-most columns show the input image and ground truth. The next on the right shows the image with the predicted bounding boxes. The green ones indicate the joint regions without special findings, while the blue or red ones indicate those with special findings. The right-most image pairs show two heat maps indicating the contributing region of the input image, with the parts contributing significantly to the output displayed in red. Although heatmaps were visualised from the Conv 7 and 9-2 layers in this study, they could be visualised from any layer in the SSD.

The overall trend, similar to that of the ankylosis and subluxation/dislocation findings obtained with the consent of doctors, confirmed that the DNN model strongly responded to the regions of the image used for mTSS estimation. The contribution regions between ankylosis and subluxation/dislocation classification differed. In subluxation/dislocation classification, the SSD often reacted to cover the entire joint region (heatmaps of Conv9-2, Fig. 6), whereas in ankylosis classification, it reacted strongly around the joint base (heatmaps of Conv9-2, Fig. 7). This observation is consistent with that obtained through the original diagnostic process for special findings. Therefore, the proposed model correctly recognised the image features required to determine the mTSS.

In the failed detection and classification cases, the SSD may not be able to detect uneven bounding boxes at the edge of the input image (Fig. 7b). This is because these images do not contain the information required to detect the joints and their mTSSs. In some cases, the model focused on regions that were not related to the mTSS (Fig. 7d). This may be attributed to a small number of training data samples, low image resolution, and cropping size. Omitted data issues can be avoided by combining multiple slide images (Fig. 3d).

In some cases, the DNN model appears to make an erroneous prediction, although it is the doctor’s annotation (ground truth) that is incorrect. This suggests that humans may have difficulty in making good decisions in cases where the confirmation of special findings is subtle. Such instances may be avoided by reverting the prediction results of the DNN model to the doctor and obtaining improved annotated data.

### Limitations

The proposed method for an automatic radiographic scoring system using the mTSS has several limitations. First, the proposed model excluded the carpal and foot joints. This was owing to the fact that the structures of the carpal and foot joints are complex, making it difficult to conduct learning experiments using our model for special finding estimations to determine the mTSS. This aspect may be addressed by constructing a model for object segmentation, rather than object detection, using a bounding box. In addition, as the number of foot cases is small, it is difficult to apply a DNN that requires a large amount of data. To address this, we plan to apply learning techniques, such as weakly supervised learning, that can be implemented using a small amount of data.

Another challenging aspect is represented by the quality of the dataset. Owing to the characteristics of the mTSS, which compares and scores the condition before and after dosing, we observed some cases of instability in annotated labels. As this is a critical issue for the mTSS, the annotation tool must be further extended to improve its efficiency and build larger datasets than the ones used in this study. Future research is planned to integrate the mTSS prediction model and annotation tools for the development of a system that provides continuous data supply and diagnostic assistance.

### Future outlooks

We have already developed a method for calculating scores that takes into account the mTSS at two time points^27^, and we plan to combine our method with the results of the present study to develop a more accurate algorithm for automatic assessment of mTSS. Assessing the degree of joint destruction, such as mTSS, is complicated and time-consuming and has rarely been used in conventional real-world clinical practice. However, if this evaluation method can be automated, the degree of joint destruction in patients can be assessed in detail simply by taking X-rays, which will not only simplify the evaluation of drug efficacy in clinical trials, but also help to recognise joint destruction at an early stage in real-world clinical practice, which will be useful in formulating patient treatment strategies.

## Conclusion

This paper proposed a model that detects the PIP/IP and MP joint regions of the hand on X-ray images and classifies them using ankylosis and subluxation labels. As training and test datasets, 260 X-ray images were prepared and annotated using an extended annotation tool for RA. Highly accurate detection performance was achieved through learning experiments and by introducing an ensemble mechanism that uses multiple detectors for each special finding. Additionally, we visualised the contribution regions of the proposed detection model using GradCAM, confirming that it appropriately captured the features of the regions where special findings were found. In future, we plan to combine our mTSS with those reported previously^27^ to automatically estimate the erosion and joint space narrowing score for diagnostic purposes.

## Data Availability

The data underlying this article cannot be shared publicly due to the privacy of individuals that participated in the study. The data will be shared by reasonable request to the lead contact. This paper does not report original code. Any additional information required to reanalyze the data reported in this paper is available from the lead contact (izz@keio.jp) upon request.
